# Patients With Mild ADPKD by Kidney Imaging but Low Estimated GFR

**DOI:** 10.1016/j.ekir.2025.03.045

**Published:** 2025-04-03

**Authors:** Seung Heyck Lee, Mauricio Miranda Cam, Taher Dehkharghanian, Fatemah Nasri, Saima Khowaja, Amirreza Haghighi, Xuewen Song, Korosh Khalili, York Pei

**Affiliations:** 1 Division of Nephrology, University Health Network and University of Toronto, Toronto, Ontario, Canada; 2 Department of Medical Imaging, University Health Network and University of Toronto, Toronto, Ontario, Canada; 3 Division of Genetics, Brigham and Women’s Hospital and Harvard Medical School, Boston, Massachusetts, USA

**Keywords:** ADPKD, biomarkers, CKD, patient-centered care, polycystic kidney disease, renal function

## Abstract

**Introduction:**

High height-adjusted total kidney volume (HtTKV) and low estimated glomerular filtration rate (eGFR) typically indicate high cystic burden by imaging and low kidney function, respectively, identifying high-risk patients for disease progression in autosomal dominant polycystic kidney disease (ADPKD). Here, we report the prevalence, clinical characteristics, and causes of mild ADPKD in patients using imaging and low eGFR, an ill-defined clinical scenario.

**Methods:**

We studied 473 patients with kidney function measurements, *PKD1* and *PKD2* genetic screen, and total kidney volume (TKV) measurements by magnetic resonance imaging (MRI) or computed tomography. Mayo Clinic Imaging Classification (MCIC) based on age and HtTKV was used to assess cystic disease severity. Patients with a discordant phenotype were defined as those with MCIC 1A/B and eGFR < 80 ml/min per 1.73 m^2^. We reviewed medical records to compare patients with and without the discordant phenotype, examining clinical characteristics such as second kidney disease(s), nephrotoxic exposure, diabetes mellitus, and metabolic syndrome-related traits.

**Results:**

Of 473 patients, 55 (12%) displayed a discordant phenotype. Among these patients, 13 (24%) had normal kidney functions by 24-h creatinine clearance (CrCl_24_) (> 80 ml/min per 1.73 m^2^) and high urinary creatinine excretion rates, indicating underestimation of their kidney function by eGFR likely because of high muscle mass. In addition, discordant patients showed a higher prevalence of hypertension (82% vs. 57%, *P* < 0.001), dyslipidemia (58% vs. 15%, *P* < 0.001), diabetes mellitus (15% vs. 3%, *P* < 0.05), and a second kidney disease (16% vs. 1%, *P* < 0.001).

**Conclusion:**

Mild ADPKD by imaging with low eGFR represents a significant clinical scenario with conflicting prognostic indicators, underscoring the need for delineating underlying causes and providing more appropriate management.

## Graphical abstract

**Figure:**
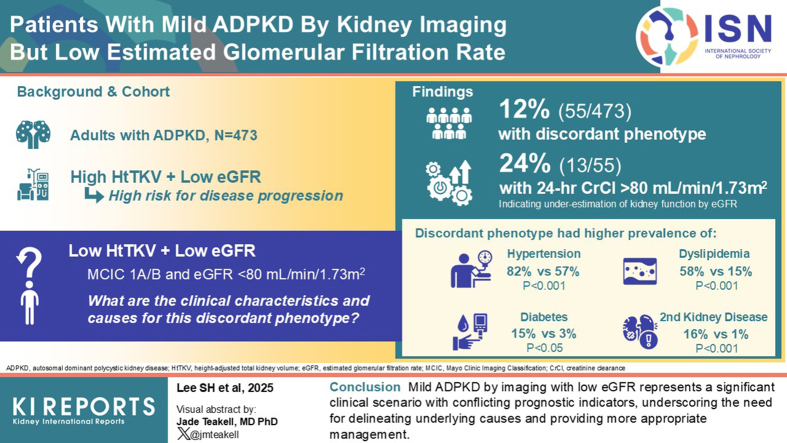

ADPKD is the most commonly inherited kidney disease worldwide and the fourth leading cause of end-stage kidney disease in North America.^1^, ^2^, ^3^ Mutations in 2 genes, *PKD1* and *PKD2*, account for most of the clinically ascertained cases. Distortion of kidney architecture from progressive increase in cyst number and size ultimately lead to chronic kidney disease (CKD) in many patients and accounts for 5% to 10% of end-stage kidney disease in developed countries.^4^ Kidney function test such as eGFR typically is within the normal range in most patients with ADPKD during the early to midstage of their clinical course, and only declines in mid- to late stages when a critical kidney size is reached.^4^ The natural course of CKD progression in ADPKD is highly variable between patients but is influenced by multiple factors such as age, sex (biological male/female), mutation class, and presence of early onset hypertension or urologic complications.^5^^,^^6^

With the approval of tolvaptan as the first disease-modifier drug for ADPKD, identifying patients at high risk for progression who may benefit from this treatment is an important clinical priority.^7^^,^^8^ The Consortium for Radiologic Imaging Studies of Polycystic Kidney Disease (PKD) has shown that TKV in ADPKD expands quasi-exponentially during adult life at approximately 5%/yr.^9^ Using age-adjusted TKV and HtTKV, the MCIC provides a validated approach for selecting “high-risk” patients, defined as class 1C-1E, for clinical trials.^10^^,^^11^ By contrast, the European Renal Association Working Group in Inherited Kidney Disease and the European Rare Kidney disease reference network employed an approach that is based on eGFR indexed for age and its rate of decline for risk assessment for ADPKD.^12^ Since 2015, we have routinely used MCIC and eGFR for risk assessment of patients with ADPKD at the Center for Innovative Management of PKD (www.cimpkd.ca) in Toronto, and we noticed that some patients with mild disease identified by kidney imaging unexpectedly had reduced eGFR. Here, we report a systematic study to define the prevalence, clinical characteristics, and causes of this discordant phenotype in patients with ADPKD, a scenario of conflicting clinical findings which has not been well-defined.

## Methods

### Study Population

**Figure 1:**
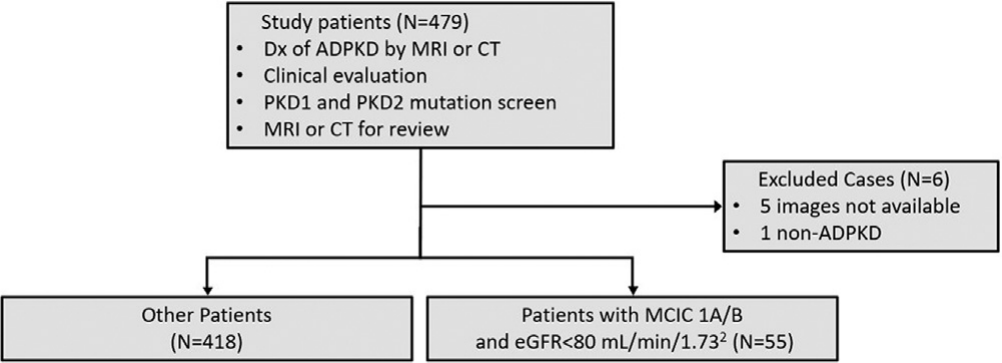
Flow chart for study patients. eGFR, estimated glomerular filtration rate; MCIC, Mayo Clinic Imaging Classification.

In Figure 1, we show a study flow diagram for patient selection. A total of 479 patients with putative ADPKD without atypical kidney imaging designation (MCIC 2) were recruited into the extended Toronto Genetic Epidemiology Study of PKD between March 1, 2016 and September 30, 2018 and followed-up with for 6.7 (± 0.6) years at the Center for Innovative Management of PKD at the University Health Network in Toronto. Five patients whose kidney imaging studies were not available and 1 patient who did not have ADPKD were excluded from further analysis. The study cohort comprised 473 patients, aged ≥ 18 years, diagnosed with ADPKD using renal MRI or computed tomography, and/or genetic testing. None of the study patients had previous kidney cyst sclerotherapy or received tolvaptan treatment before their MRI. They were referred by more than 100 academic and community nephrologists in the Greater Toronto Area for risk assessment by genetic testing and kidney MRI, and potential therapeutic interventions. The study was approved by the Ethics Review Board at the University Health Network in Toronto and conducted in accordance with the Declaration of Helsinki. All study patients provided written informed consent.

### Exposure and Outcomes

We employed a retrospective cross-sectional study design to determine the prevalence of disease discordant patients by eGFR and HtTKV at presentation during their first MRI or computed tomography date. eGFR was calculated using the CKD-Epidemiology Collaboration equation.^13^ Kidney volume was measured using MRI or computed tomography using the ellipsoid method and read by author FN. Using age-adjusted TKV and HtTKV, each patient was assigned an MCIC.^10^ Patients with MCIC 1A or 1B were considered as “low risk,” MCIC 1C were considered as “intermediate risk,” and MCIC 1D or 1E were considered as “high risk” for progression to end-stage kidney disease. Demographic and follow-up clinical outcome data including age, sex, height, weight, hematuria, proteinuria, blood pressure control and treatment, lipid profile and treatment, smoking and alcohol use, presence of diabetes mellitus, nephrotoxic exposure, or a second kidney disease were extracted from all patients (by SHL, MMC, and TD). Additional data on kidney function measurements, including serum creatinine, 24-h urine creatinine excretion rate and creatinine clearance within 5 months of kidney imaging were collected from all study patients. Longitudinal follow-up data were collected as part of the discordant phenotype workup (i.e., progression of CKD stage, kidney biopsies, and genetic studies).

Genetic screening for *PKD1* and *PKD2* mutations was performed using targeted exome sequencing, and all pathogenic mutations were confirmed by Sanger sequencing using a validated polymerase chain reaction protocol.^5^ All nonsense, frameshift, and canonical splice site mutations were grouped as protein-truncating (PT) mutations, and nonsynonymous missense or atypical splice site mutations were grouped as nontruncating mutations. In-frame insertions or deletions (in-frame indel) were classified separately. All nontruncating mutations were evaluated for their pathogenicity using prediction algorithms (Align GVGD, PolyPhen-2, SIFT, PROVEAN, and Human Splicing Finder), review of the PKD mutation database (http://pkdb.mayo.edu), and by segregation analysis with additional affected family members when possible. In addition, all mutation-negative patients were screened by multiplex ligation-dependent probe amplification to detect large gene arrangements.^5^

### Statistical Analysis

All collected parameters were compared between the study patients with and without disease discordance (MCIC 1A and 1B and eGFR < 80 ml/min per 1.73 m^2^). Subgroup analysis of MCIC 1A and 1B patients was also performed, comparing those with eGFR < 80 ml/min per 1.73 m^2^ and eGFR > 80 ml/min per 1.73 m^2^. Normally distributed continuous variables were expressed as mean ± SD, nonnormal continuous variables as median (interquartile range, IQR), and categorical variables as frequency (percentage). Patient characteristics were compared using the Mann-Whitney test and Fisher exact test. For patients with available kidney function measurements, eGFR and CrCl_24_ were compared in pairs using Wilcoxon signed-rank test. Age, sex, body mass index, and mutation category (PKD1 PT/indel vs. all others) were adopted for multiple linear regression analysis of independent risk factors for eGFR in the MCIC 1A and 1B subgroup. Correlation between age and eGFR was analyzed using Pearson’s correlation test. All tests were 2-sided and a *P*-value < 0.05 was considered statistically significant. Statistical analysis was performed in R (v4.0.3, R Foundation for Statistical Computing, Vienna, Austria) and GraphPad v8.4.3 (San Diego, CA).

## Results

**Figure 2:**
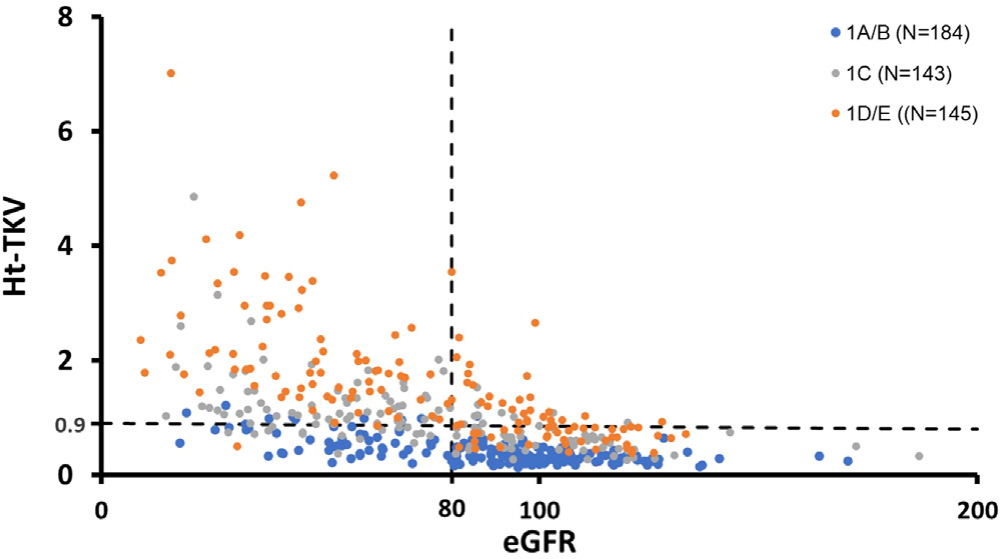
Distribution of Mayo Clinic Imaging Class (MCIC) based on height-adjusted total kidney volume (HtTKV in l/m) and estimated glomerular filtration rate (eGFR in ml/min per 1.73 m^2^). Using HtTKV < 0.9 l/m and eGFR < 80 ml/min per 1.73 m^2^, excluding those with MCIC 1C/D/E, 55 of 473 patients (12%) were found to have mild disease by kidney imaging (MCIC 1A or 1B) and yet low eGFR < 80 ml/min per 1.73 m^2^. eGFR, estimated glomerular filtration rate.

In Figure 2, we show the distribution of study patients by their HtTKV and eGFR cut-offs. Most patients with HtTKV ≥ 0.9 l/m and eGFR ≥ 80 ml/min per 1.73 m^2^ and HtTKV ≥ 0.9 l/m and eGFR < 80 ml/min per 1.73 m^2^ have early and later stages of moderate to severe PKD, respectively; their disease severity is concordant based on their kidney imaging and eGFR. By contrast, most patients with HtTKV < 0.9 l/m and eGFR ≥ 80 ml/min per 1.73 m^2^ have mild (MCIC 1A or 1B), moderate (MCIC 1C), or severe (MCIC 1D-1E) PKD at a younger age. Of interest, 12% (55/473) of the study patients displayed a discordant phenotype (i.e., MCIC 1A or 1B and eGFR < 80 ml/min per 1.73 m^2^) with most, but not all in the grouping with mild PKD by kidney imaging but low eGFR (i.e., HtTKV < 0.9 l/m and eGFR < 80 ml/min per 1.73 m^2^).

The clinical characteristics of patients with discordant versus concordant phenotype are shown in Table 1. Compared with the concordant patients (*n* = 418), discordant patients were older (62 vs. 41 years, *P* < 0.001); however, there was no significant differences in sex (female: 53% vs. 56%, *P* = NS) and body mass index (median [IQR]: 26 [23–29] vs. 26 [23–30] kg/m^2^, *P* = 0.87) between the 2 groups. Discordant patients were less likely to have *PKD1* PT mutations (18% vs. 40%, *P* < 0.001), but more likely to have *PKD2* mutations (38% vs. 11%, *P* < 0.001), or no *PKD1/PKD2* mutations detected (NMD) (27% vs. 11%, *P* < 0.001). Of the 15 discordant patients with NMD results, only 4 had a family history of ADPKD, whereas secondary genetic analysis by multiplex ligation-dependent probe amplification confirmed 8 NMD cases and 7 cases with one of the following: *COL4A1, COL4A3, COL4A4, UMOD, UGGT2, and ALG8*. None of the patients with NMD had a detectable *DNAJB11* mutation. Discordant patients also showed significant higher rates of proteinuria (51% vs. 11%, *P* < 0.001). Discordant patients had significantly more comorbid conditions of hypertension (82% vs. 57%, *P* < 0.001), dyslipidemia (58% vs. 15%, *P* < 0.001), diabetes mellitus (15% vs. 3%, *P* < 0.01), and a second kidney disease (16% vs. 1%, *P* < 0.001). Lifestyle histories of smoking (20% vs. 20%, *P* = 1) and regular use of alcohol (31% vs. 26%, *P* = 0.41) were not significantly different between the 2 groups.

**Table 1:** Characteristics of patients with and without MCIC 1A-1B and eGFR<80 ml/min per 1.73 m^2^ at present or during follow-up

Clinical characteristics	MCIC 1A–1B and eGFR < 80 ml/min per 1.73 m^2^	
	Yes (*n* = 55)	No (*n* = 418)
Age (yrs)^a^	62 (54–68)	41 (32–51)
Female sex	29 (53)	234 (56)
BMI (kg/m^2^)^a^	26 (23–29)	26 (23–30)
Mutation class		
PKD1 PT	10 (18)	169 (40)
PKD1 in-frame indel	1 (2)	17 (4)
PKD1 NT	8 (15)	80 (19)
PKD2	21 (38)	47 (11)
NMD	15 (27)	45 (11)
eGFR (ml/min per 1.73 m^2^)^b^	56 (43–65)	88 (61–105)
CrCl (ml/min per 1.73 m^2^)^c^	70 (55–83)	95 (67–118)
HtTKV (ml/m)	571 (430–812)	727 (377–1298)
Urinalysis		
Hematuria	7 (13)	26 (6)
Proteinuria	28 (51)	45 (11)
MCIC		
1A–B	55 (100)	130 (31)
1C	0 (0)	143 (34)
1D–E	0 (0)	145 (35)
Follow-up duration (yrs)	6.7 (6–7.1)	6.9 (6.4–7.3)
Clinical history		
Hypertension	45 (82)	240 (57)
Dyslipidemia	32 (58)	63 (15)
Diabetes	8 (15)	13 (3)
Second kidney disease	9 (16)	3 (1)
Lifestyle history		
Current or exsmoker	11 (20)	85 (20)
Regular alcohol use	17 (31)	108 (26)

BMI, body mass index; CT, computed tomography; eGFR, estimated glomerular filtration rate; HtTKV, height-adjusted total kidney volume; MCIC, Mayo Clinic Imaging Class; MRI, magnetic resonance imaging; NMD, no mutation detected; NT, nontruncating; PT, protein-truncating.

Data expressed as number (%) or median (interquartile range). *P*-values obtained using Fisher exact test and Mann-Whitney tests.

a Missing BMI: 1 from Yes and 26 from No group.

b At MRI or CT scan.

c Missing CrCl_24_ (24-h creatinine clearance): 1 from Yes and 39 from No group.

**Figure 3:**
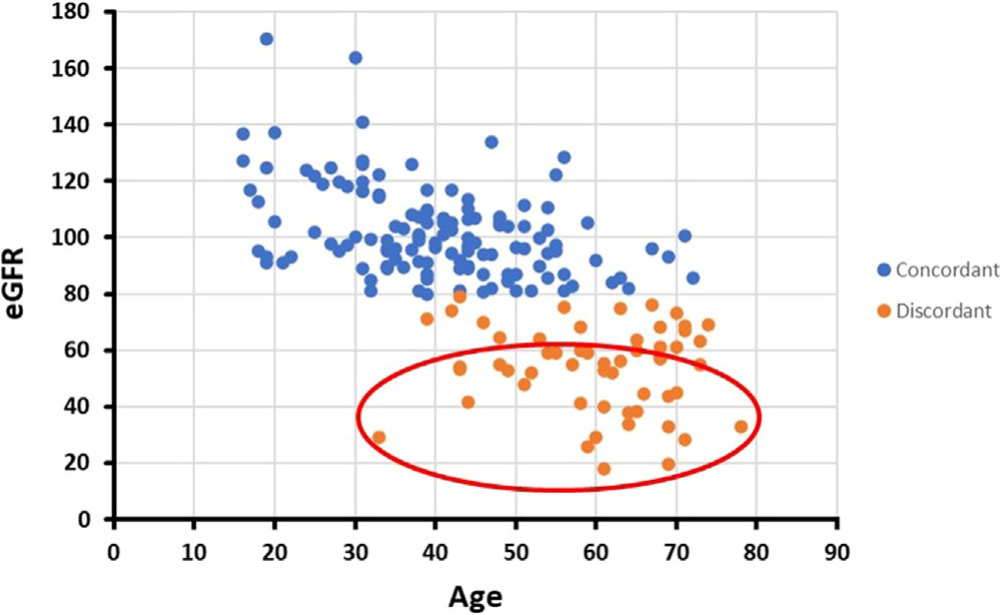
Distribution of patients with Mayo Clinic Imaging Class (MCIC) 1A/B based on age (years) and estimated glomerular filtration rate (eGFR in ml/min per 1.73 m^2^). Multiple linear regression showed that age is a significant predictor for eGFR decline (*P* < 0.001) with a negative Pearson’s correlation (*R* = −0.65, *R*^*2*^ = 0.43). Among the discordant patients, a subset (denoted within the red oval) displayed more reduced eGFR than would be expected by their age-related changes and ADPKD phenotype, raising the possibility of additional factors contributing to the disease variability. ADPKD, autosomal dominant polycystic disease; eGFR, estimated glomerular filtration rate.

In Figure 3, we show the distribution of patients with MCIC 1A and 1B by their age and eGFR. Multiple linear regression showed that age was a significant predictor of eGFR decline (β: −1.26, 95% confidence interval: [−1.50 to −1.02], *P* < 0.001) with a negative Pearson’s correlation (*R* = −0.65, *R*^*2*^ = 0.43, *P* < 0.001), but not sex, body mass index, or mutation category (*P* > 0.05). Subgroup comparison MCIC 1A and 1B patients with eGFR < 80 ml/min per 1.73 m^2^ (disease discordant) and eGFR > 80 ml/min per 1.73 m^2^ are shown in Table 2. Discordant patients were significantly older (62 vs. 39 years, *P* < 0.001) and had higher rates of hypertension (82% vs. 32%, *P* < 0.001), dyslipidemia (58% vs. 10%, *P* < 0.001), diabetes (15% vs. 4%, *P* < 0.05), and second kidney disease (16% vs. 0%, *P* < 0.001). They had atypical severity cases (16% vs. 6%, *P* < 0.05) but similar rates of NMD (27% vs. 16%, *P* > 0.05). Among the discordant patients, a subset (denoted within the red oval) displayed more reduced eGFR than would be expected by their age-related changes and ADPKD phenotype (Figure 3), raising the possibility of additional factors contributing to the disease variability.

**Table 2:** Characteristics of patients with and without MCIC 1A–1B and eGFR < 80 ml/min per 1.73 m^2^ at present or during follow-up

Clinical characteristics	MCIC 1A–1B	
	eGFR < 80 (*n* = 55)	eGFR > 80 (*n* = 130)
Age (yrs)^a^	62 (54–68)	39 (32–49)
Female sex	29 (53)	79 (61)
BMI (kg/m^2^)^a^	26 (23–29)	24 (21–30)
Mutation class		
PKD1 PT	10 (18)	29 (22)
PKD1 in-frame indel	1 (2)	6 (5)
PKD1 NT	8 (15)	30 (23)
PKD2	21 (38)	44 (34)
NMD	15 (27)	21 (16)
eGFR (ml/min per 1.73 m^2^)^b^	56 (43–65)	99 (90–110)
CrCl (ml/min per 1.73 m^2^)^c^	70 (55–83)	114 (97–131)
HtTKV (ml/m)	571 (430–812)	318 (244–389)
Urinalysis		
Hematuria	7 (13)	9 (6)
Proteinuria	28 (51)	6 (5)
Follow-up duration (yrs)	6.7 (6–7.1)	6.9 (6–7.3)
Clinical history		
Hypertension	45 (82)	42 (32)
Dyslipidemia	32 (58)	13 (10)
Diabetes	8 (15)	5 (4)
Second kidney disease	9 (16)	0 (0)
Lifestyle history		
Current or exsmoker	11 (20)	23 (18)
Regular alcohol use	17 (31)	35 (27)

BMI, body mass index; CT, computed tomography; eGFR, estimated glomerular filtration rate; HtTKV, height-adjusted total kidney volume; MCIC, Mayo Clinic Imaging Class; MRI, magnetic resonance imaging; NMD, no mutation detected; NT, nontruncating; PT, protein-truncating.

Data expressed as number (%) or median (interquartile range). *P-*values obtained using Fisher exact test and Mann-Whitney tests.

a Missing BMI: 1 from eGFR < 80 and 15 from eGFR > 80 group.

b At MRI or CT scan.

c Missing CrCl_24_ (24-h creatinine clearance): 1 from eGFR < 80 and 20 from eGFR > 80 group.

**Figure 4:**
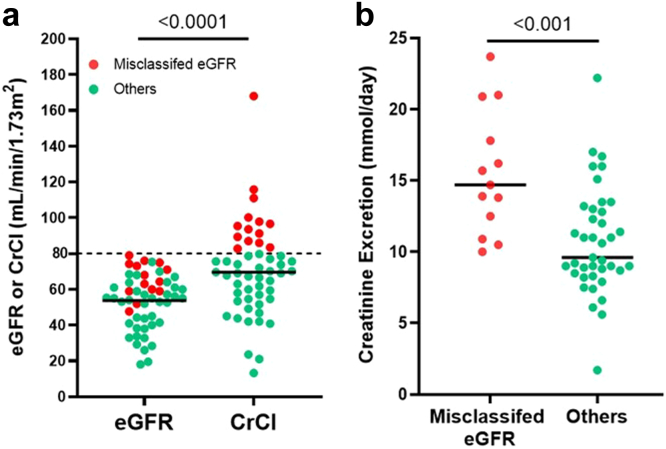
Kidney function measures of patients with disease discordance. (a) Comparison of estimated glomerular filtration rate (eGFR) on MRI date using 2012 CKD-EPI equation and 24-h urine collection. eGFR > 80 indicates relatively healthier kidneys. Of the discordant patients 24% (13/55) had misclassified kidney function according to eGFR measurements, where CKD-EPI eGFR < 80 ml/min per 1.73 m^2^ and CrCl > 80 ml/min. CrCl was significantly higher than eGFR (*P* < 0.0001). (b) Creatinine excretion rate of patients with misclassified eGFR was significantly higher (*P* < 0.001) than other patients with nonmisclassified eGFR. CrCl and creatinine excretion were unavailable from 1 patient. Note: 1 patient with misclassified eGFR had a second kidney disease. CKD-EPI, Chronic Kidney Disease-Epidemiology Collaboration equation; CrCl, creatinine clearance; MRI, magnetic resonance imaging.

Upon review of their medical records, we found that discordant patients can be grouped into those with the following: (i) misclassified eGFR, (ii) a second kidney disease, or (iii) no apparent cause of discordance. The clinical characteristics of the discordant patients under these groupings are shown in Table 3. Overall, eGFR was lower than measured CrCl_24_ in the discordant patients (i.e., median [IQR]: 56 [43–65] vs. 70 [55–83] ml/min per 1.73 m^2^, *P* < 0.001; Table 1) with a median difference of 16 ml/min per 1.73 m^2^ (approximately 24% difference) and 13 of 55 (24%) of the discordant patients had CrCl_24_ significantly higher than 80 ml/min per 1.73 m^2^ with 30% difference on average (i.e., median [IQR]: 64 [59–74] vs. 94 [87–104] ml/min per 1.73 m^2^; Figure 4a). Discordant patients with misclassified eGFR (compared with CrCl_24_) also showed significantly higher 24-h creatinine excretion rate than discordant patients with correctly classified glomerular filtration rate (GFR) (median [IQR]: 15 [12–19] vs. 10 [9–13] mmol/d, *P* < 0.01; Figure 4b). By contrast, the eGFR of the concordant patients was also lower than CrCl_24,_ but with a smaller median difference of 6 ml/min per 1.73 m^2^ (median [IQR]: 88 [61–105] vs. 95 [67–117] ml/min, *P* < 0.001; Table 1). We found that 9 of 55 discordant patients (16%) were diagnosed with at least 1 second kidney disease in addition to ADPKD; their clinical characteristics are shown in Table 4. Abdominal imaging results are shown in Supplementary Figure S1. The second kidney diseases include focal segmental glomerulosclerosis, renal parenchymal atrophy, diabetic nephropathy, thin basement membrane disease, renal cell carcinoma with partial nephrectomy, and analgesic nephropathy from chronic nonsteroidal antiinflammatory drug use. All these patients showed low risk of progression to end-stage renal disease based on MCIC 1A–1B, but moderate to severe loss in kidney function based on CKD stages (3 patients had CKD stage 4, 5 had CKD stage 3A/B, and 1 had CKD stage 2). Their mean GFR and CrCl_24_ were 48 and 58 ml/min per 1.73 m^2^, respectively, and their mean proteinuria was 1.0 g/d. Compared with the concordant patients and MCIC 1A/B patients with eGFR > 80 ml/min per 1.73 m^2^, discordant patients were enriched with hypertension, dyslipidemia, and diabetes mellitus (Table 1, Table 2). However, there were no significant differences for enrichment of these risk factors among the 3 discordant subgroups except for a higher prevalence of dyslipidemia and diabetes mellitus in the discordant patients with a second kidney disease and a lower prevalence of smokers in the discordant patients with misclassified eGFR (Table 3).

**Table 3:** Characteristics of patient subgroups with mild PKD by kidney imaging and low eGFR

Characteristics	Misclassified eGFR^a^ (*n* = 13)	Second kidney disease^b^ (*n* = 9)	Others (*n* = 33)
Age (yrs)^c^	55 (46–66)	61 (53–64)	65 (58–69)
Female sex	8 (62)	4 (44)	17 (51)
BMI (kg/m^2^)	27 (22–31)	27 (26–29)	25 (23–27)^d^
Mutation class			
PKD1 PT	2 (15)	2 (22)	6 (18)
PKD1 in-frame indel	0 (0)	0 (0)	1 (3)
PKD1 NT	3 (23)	1 (11)	3 (9)
PKD2	5 (38)	3 (33)	12 (36)
NMD	3 (23)	3 (33)	11 (33)
eGFR (ml/min per 1.73 m^2^)^c^	64 (59–74)	53 (34–64)	55 (41–61)
Creatinine clearance (ml/min)^c^	94 (87–104)	53 (42–76)	64 (51–72)^e^
HtTKV (ml/m)^c^	573 (366–710)	558 (473–620)	581 (435–871)
Urinalysis			
Hematuria	1 (8)	2 (22)	3 (9)
Proteinuria	6 (43)	6 (67)	16 (50)
Follow-up duration (yrs)	6.6 (6.3–7.3)	6.9 (6–7)	6.7 (5.9–7.1)
Clinical history			
Hypertension	11 (85)	8 (89)	26 (79)
Dyslipidemia	6 (46)	8 (89)	18 (55)
Diabetes	2 (15)	2 (22)	4 (12)
Second kidney disease	0 (0)	9 (100)	0 (0)
Lifestyle history			
Current or exsmoker	1 (8)	3 (33)	7 (21)
Regular alcohol	3 (23)	4 (44)	10 (30)

BMI, body mass index; CrCl, creatinine clearance; eGFR, estimated glomerular filtration rate; HtTKV, height-adjusted total kidney volume; MCIC, Mayo Clinic Imaging Class; MRI, magnetic resonance imaging; NMD, no mutation detected; NT, nontruncating; PKD, polycystic kidney disease; PT, protein-truncating.

Data expressed as number (%) or median (interquartile range). Two patients with missing CrCl excluded. *P*-values obtained using Fisher exact test and Mann-Whitney tests.

a Patients with HtTKV < 900, eGFR < 80 and CrCl > 80.

b One patient with a second kidney disease also had misclassified eGFR.

c At MRI scan.

d Missing BMI for 1 patient.

e Missing CrCl for 1 patient.

**Table 4:** Characteristics of discordant patients with mild ADPKD by kidney imaging and low eGFR who were found to have a second kidney disease (*n* = 9)

Pt. ID	Age^a^ (yrs)	Sex	Mutation	MCIC	CKD stage^a^	eGFR^a^	CrCl^a^	Proteinuria^a^	Second kidney disease
P1	33	F	PKD1 PT	1B	4	29	37	Negative	Increased global glomerulosclerosis, abnormal glomerular basement membrane suggestive of spectrum of TBMD and Alport syndrome (AS)^b^
P2	43	F	PKD1 NT	1B	3A	52	53	0.5	TBMD with mild tubular atrophy^c^
P3	53	M	PKD2	1B	3A	64	76	2.6	Focal segmental glomerulosclerosis (FSGS) with arterial nephrosclerosis and vascular disease^b^
P4	61	M	NMD confirmed	1B	4	53	77	0.2	Left partial nephrectomy for renal cell carcinoma, previous history of acute kidney injury
P5	61	F	PKD1 PT	1B	4	18	21	Negative	Mild global glomerulosclerosis, many shrunken glomeruli, interstitial fibrosis^b^
P6	64	M	PKD2	1B	3B	34	52	Negative	Unclear clinical picture of atypical renal parenchymal atrophy^c^
P7	64	M	NMD confirmed	1A	3B	38	67	1.4	Diabetic nephropathy, hypertensive nephrosclerosis^b^
P8	67	M	NMD/COL4A3	1B	2	76	100	1.4	Type 2 diabetes mellitus, FSGS, TBMD^b^
P9	71	F	PKD2	1B	3A	67	42	0.05	Chronic NSAID usage for 6 years with possible analgesic nephropathy

ADPKD, autosomal dominant polycystic disease; CKD, chronic kidney disease; CrCl, creatinine clearance; CT, computed tomography; eGFR, estimated glomerular filtration rate; F, female; M, male; MCIC, Mayo Clinic Imaging Classification; MRI, magnetic resonance imaging; NMD, no mutation detected; NSAID, nonsteroidal antiinflammatory drug; PT, protein-truncating; Pt., patient; TBMD, thin basement membrane disease.

Abdominal images are included in Supplementary Figure S1.

a At MRI date.

b Diagnosed with renal biopsy.

c Diagnosed with CT.

## Discussion

The Consortium for Radiologic Imaging Studies of PKD has shown that kidney volume expands approximately 5%/yr during adult life in ADPKD.^14^ Using age-adjusted TKV and HtTKV obtained from MRI, MCIC provides a validated biomarker to identify high-risk patients for CKD progression.^10^^,^^11^ In addition, eGFR is a convenient biomarker for assessing kidney function and when indexed for specific age range, which has also been used for CKD risk assessment in ADPKD.^12^ In this large and clinically well-characterized cohort, we observed that 12% (55/473) of patients with MCIC 1A–1B had eGFR < 80 ml/min per 1.73 m^2^, a discordant phenotype of kidney disease severity between imaging and functional testing. We further delineated these discordant patients into 3 subgroups, including those with the following: (i) misclassified eGFR, (ii) a second kidney disease, or (iii) no apparent cause of discordance.

The discordance between various kidney function measurements and the imperfect nature of serum creatinine–based measurements has been extensively studied in the past few decades.^15^ In clinical practice, creatinine is the most widely available endogenous marker for GFR estimation because it is freely filtered across the glomerulus without significant tubular reabsorption, except at low GFRs. Serum creatinine is routinely measured from blood tests and estimates GFR using various formulas that account for age, sex, and body weight.^15^ However, muscle mass is an important source of error in serum creatinine–based GFR estimation.^16^ Because creatinine is metabolized from creatine, which is released by the muscles, patients with higher muscle mass have higher serum creatinine and thus an underestimated eGFR.^17^ We found that 24% of the discordant patients (13/55) displayed significantly higher CrCl_24_ (by almost 30% compared with their eGFR) that was well within the normal range (Figure 4a**)** and they also had a high to supranormal 24-h urinary creatinine excretion rate (median [range]: 15 [12–19] mmol/d; normal range: 5.3–16 mmol/d; Figure 4b). Taken together, these findings suggest that this subgroup of patients had misclassified kidney function based on their eGFR, most likely because of high muscle mass and/or increased dietary protein intake.^15^, ^16^, ^17^, ^18^ Further investigations by measuring 24-h creatinine excretion rate and cystatin-based eGFR may provide a more accurate delineation of their eGFR.

We found that 9 discordant patients were diagnosed with at least 1 second kidney disease that presumably explains their low eGFR; their CKD stages based on eGFR indicate moderate to severe kidney disease, which contradicts the low risk for CKD progression based on MCIC 1A–1B (Table 4). Consistent with their mild PKD burden from kidney imaging, 7 of the discordant patients had a mutation class (i.e., *PKD1* nontruncating, *PKD2*, or no mutation detected) that is typically associated with mild PKD.^5^^,^^6^ Although patients P1 and P5 had a PT *PKD1* mutation that is generally associated with severe PKD, their kidney imaging displayed mild PKD (MCIC 1B), which is reported in 18% of patients with PT *PKD1* mutations.^19^ Four patients (P1, P3, P5, and P8) were diagnosed with global or focal segmental glomerulosclerosis, a heterogeneous group of conditions with glomerular scarring due to immunologic and nonimmunological etiologies. Two of these patients (P3 and P8) presented with high-grade proteinuria, suggesting that they might have an immunological form of focal segmental glomerulosclerosis.^20^ Of note, P8 also had misclassified kidney function (eGFR: 76 ml/min per 1.73 m^2^, CrCl: 100 ml/min) at presentation, but the eGFR deteriorated to 55 ml/min per 1.73 m^2^ within a 2-year period. Three patients (P1, P2, and P8) were diagnosed with thin basement membrane disease, a relatively common (present in ∼1% of the general population) and generally benign condition associated with a heterozygous *COL4A3* or *COL4A4* mutation.^21^^,^^22^ Two patients had at least 2 separate kidney conditions (P4: partial nephrectomy and acute kidney injury; P8: type 2 diabetes mellitus, focal segmental glomerulosclerosis, and thin basement membrane disease) in addition to their PKD. P6 showed renal parenchymal atrophy on kidney biopsy but atypical ADPKD of MCIC 2 by kidney imaging, which was not appreciated at the time of study recruitment. Taken together, these cases illustrate that the presence of ≥1 separate kidney disease(s) may adversely modify the CKD severity in ADPKD.

The cause(s) for the discordant phenotype in the remaining patients are unclear but may be more nuanced. Compared with the concordant patients, these patients are older and more likely to have hypertension, dyslipidemia, and type 2 diabetes mellitus, which are comorbid risk factors of a metabolic syndrome. Poor control of these conditions may further explain the discordant phenotype but were not differentiated in this study. Recent epidemiological studies have highlighted that overweight and obesity are independent risk factors for CKD progression in ADPKD.^23^^,^^24^ In addition, renovascular disease is prevalent in the older patients with the above metabolic risk factors and often difficult to diagnose.^25^^,^^26^ In this regard, there is a growing appreciation for the pathophysiological interrelatedness of the above metabolic risk factors with cardiovascular disease as well as CKD, leading to the conceptualization of cardiovascular-kidney-metabolic syndrome,^27^ which we speculate may explain the discordant phenotype in many of the above patients. However, a lack of any validated clinical tool that captures metabolic syndrome as a complex time-dependent phenotype makes it challenging, if not impossible, to infer metabolic syndrome as the primary cause of a specific kidney disease phenotype such as CKD.

We analyzed the possible influence of age in discordant patients who were generally older by performing a MCIC 1A and 1B subgroup comparison. Age correlated partially with eGFR decline in this subgroup, meaning older age alone does not sufficiently explain the cause of disease discordance in the younger patients, especially those aged < 55 years. Although older age can account for higher prevalence of comorbid metabolic conditions and a modest reduction in eGFR in older patients, younger patients with mild ADPKD phenotype and low risk progression to end-stage renal disease would not be expected to show early pronounced eGFR decline, which would lead us to speculate about the other causes described previously.^28^ However, there is an increasing recognition that even mildly reduced eGFR in younger patients is linked to a higher risk of all-cause mortality, cardiovascular events, and kidney failure.^29^ Arguably, all these discordant patients can significantly benefit from proper kidney health screening and prescribing appropriate treatment to prevent early end-stage renal disease.

There are limitations to this study. First, we cannot eliminate referral bias because our study was based on a single PKD Specialty Center albeit with a large patient sample size and wide referral base from family physicians and community and academic nephrologists. Second, CrCl_24_ measurements were not available in 40 (study patients 8%), which could have a minor effect in our estimate of the misclassified eGFR rate (i.e., 24%) among patients with the discordant phenotype. Third, we did not have serial data to quantify and compare the duration and severity of metabolic syndrome between patients with and without the discordant phenotype.

In conclusion, discordant kidney disease severity between imaging and functional testing occurs in 12% of patients with ADPKD and requires follow-up investigations to delineate the specific cause(s). However, this clinical scenario may be missed by physicians who solely employ kidney functional data (i.e., eGFR indexed for age and/or eGFR slope) for risk assessment of ADPKD.^13^ Recognition of this clinical scenario is important for optimal management of ADPKD, including the appropriate use of tolvaptan in patients at “high-risk” for progression of their cystic kidney disease and not for other cause(s). Pending further clinical studies, the use of a sodium-glucose transport protein 2 inhibitor and/or glucagon-like peptide 1 receptor agonist may provide an effective therapy for patients with PKD whose kidney disease discordance is strongly linked to cardiovascular-kidney-metabolic syndrome.

## Disclosure

YP has received compensation for participation in advisory boards for Otsuka, Maze Therapeutic, and Sanofi-Genzyme. All the other authors declared no competing interests.

## Supplementary Material

Supplementary File (PDF) **Figure S1.** Abdominal MRI/CT of discordant patients with mild ADPKD by kidney imaging and low eGFR who were found to have a second kidney disease (*n* = 9).

## Footnotes

Supplementary File (PDF)
**Figure S1.** Abdominal MRI/CT of discordant patients with mild ADPKD by kidney imaging and low eGFR who were found to have a second kidney disease (*n* = 9).
