# Supplementary material for: Patients With Mild ADPKD by Kidney Imaging but Low Estimated GFR
Source: Kidney Int Rep. 2025 Apr 3;10(6):1855–63. doi: 10.1016/j.ekir.2025.03.045 (PMC12231023; doi:10.1016/j.ekir.2025.03.045)

Figure S1: Abdominal MRI/CT of discordant patients with mild ADPKD by kidney imaging and low eGFR who were found to have a second kidney disease (n=9).

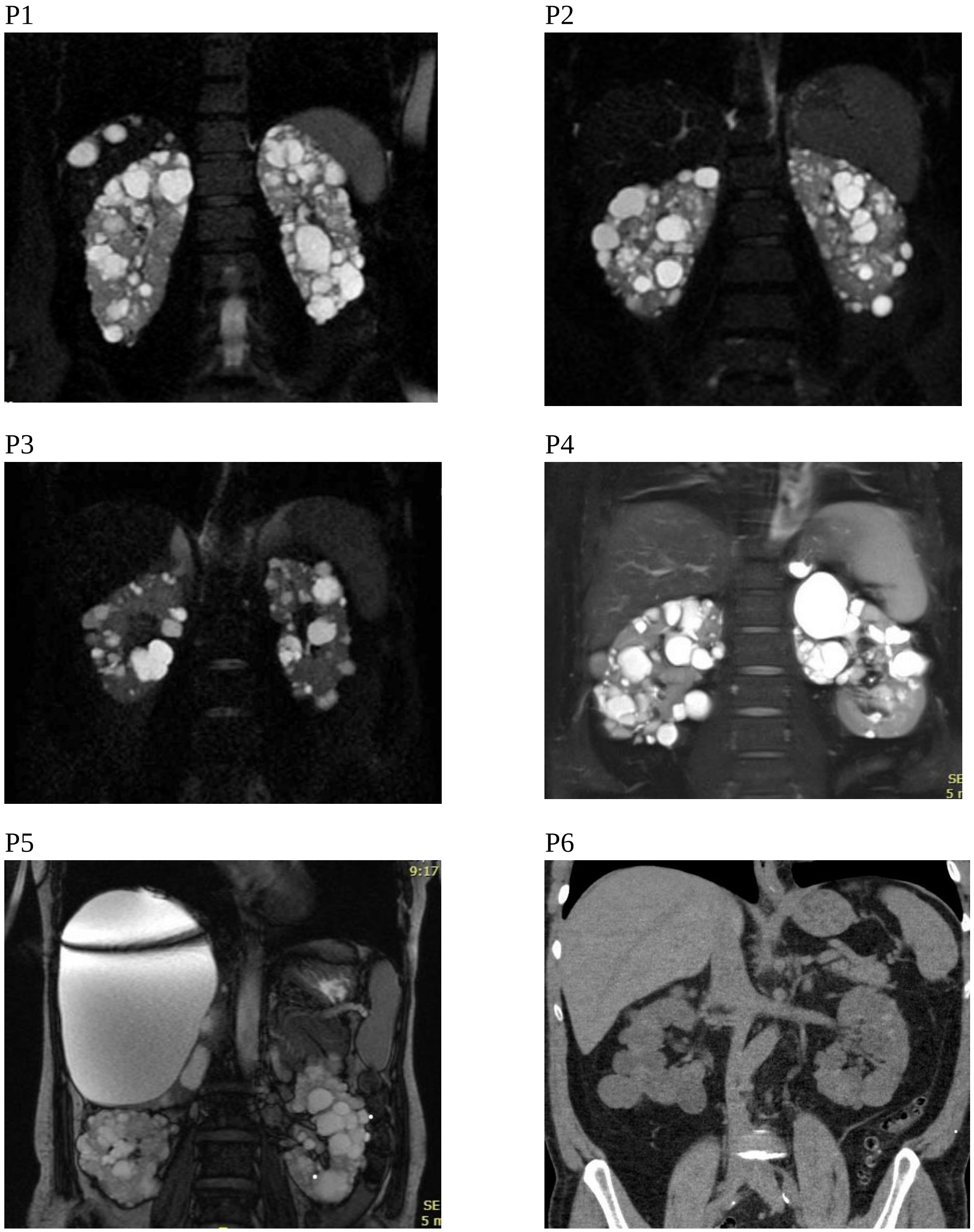

P7

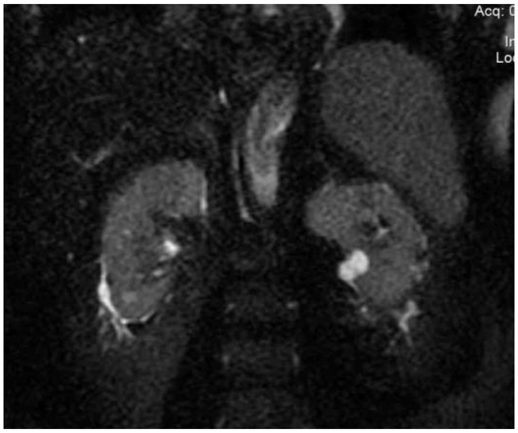

P8

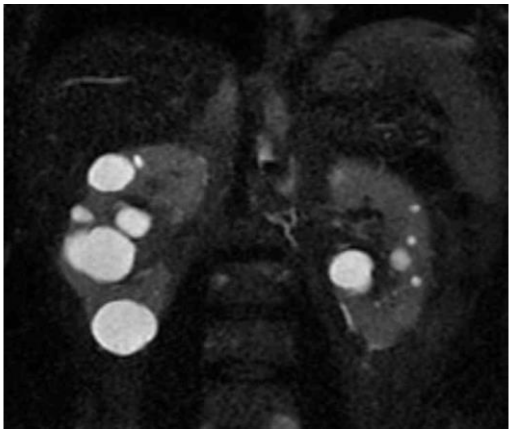

P9

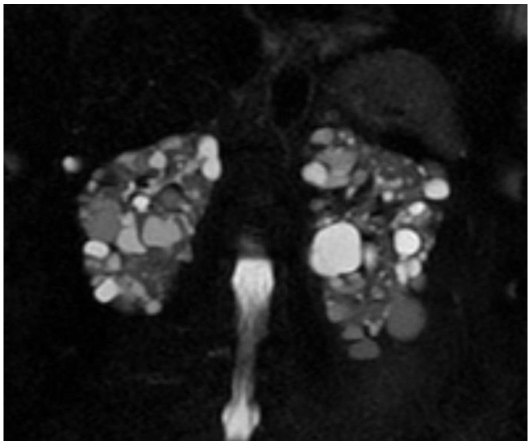

Supplement: Supplementary File (PDF) — Figure S1. Abdominal MRI/CT of discordant patients with mild ADPKD by kidney imaging and low eGFR who were found to have a second kidney disease (n = 9). [file mmc1.pdf]
